# The Vascular Endothelial Growth Factors-Expressing Character of Mesenchymal Stem Cells Plays a Positive Role in Treatment of Acute Lung Injury* In Vivo*

**DOI:** 10.1155/2016/2347938

**Published:** 2016-05-24

**Authors:** Yi Yang, Shuling Hu, Xiuping Xu, Jinze Li, Airan Liu, Jibin Han, Songqiao Liu, Ling Liu, Haibo Qiu

**Affiliations:** ^1^Department of Critical Care Medicine, Zhongda Hospital, School of Medicine, Southeast University, No. 87 Dingjiaqiao Road, Nanjing, Jiangsu 210009, China; ^2^School of Medicine, Southeast University, No. 87 Dingjiaqiao Road, Nanjing, Jiangsu 210009, China

## Abstract

Recently, mesenchymal stem cells (MSC) have been proved to be beneficial in acute respiratory distress syndrome (ARDS). Vascular endothelial growth factor (VEGF) is an important angiogenesis factor that MSC release. However, the precise role of VEGF-expressing character of MSC in the MSC treatment for ARDS remains obscure. Here, we firstly knocked down the gene VEGF in MSC (MSC-ShVEGF) with lentiviral transduction. Then we injected the MSC-ShVEGF to rats with lipopolysaccharide-induced acute lung injury (ALI) via the tail vein. Data showed that MSC transplantation significantly increased VEGF levels in the lung, reduced lung permeability, protected lung endothelium from apoptosis, facilitated VE-cadherin recovery, controlled inflammation, and attenuated lung injury. However, VEGF gene knockdown in MSC led to relatively insufficient VEGF expression in the injured lung and significantly diminished the therapeutic effects of MSC on ALI, suggesting an important role of VEGF-expressing behavior of MSC in the maintenance of VEGF in the lung and the MSC treatment for ALI. Hence, we conclude that MSC restores the lung permeability and attenuates lung injury in rats with ALI in part by maintaining a “sufficient” VEGF level in the lung and the VEGF-expressing character of MSC plays a positive role in the therapeutic effects of MSC on ARDS.

## 1. Introduction

Acute respiratory distress syndrome (ARDS) is among the leading causes of mortality in intensive care units (ICUs). Although there is some progress in the mechanism and treatment of ARDS, including lung-protective ventilation [1] and fluid-conservative strategies [2], as well as the application of prone positioning [3, 4] and paralytics [5], the incidence and overall mortality of ARDS have not yet been changed [6]. To date, pharmacologic therapies have not been able to improve clinical outcomes. Therefore, there is an urgent need for development of novel therapeutic strategies.

ARDS is characterized by the injury to alveolar-capillary membrane which results in the increased vascular permeability with exudation of proteinaceous fluid and migration of inflammatory cells from the lung vascular compartment into the interstitial and alveolar space [7]. Lung endothelium has been considered to provide the first and key barrier to protein and fluid flow into lung interstitial and alveolar space. Hence, the maintenance of the integrity of lung endothelium is critically important for the treatment of ARDS.

Mesenchymal stem cells (MSC) are a kind of cell type which has a multipotent differentiation potential. In addition, MSC also display a strong paracrine capacity [8]. MSC is able to secrete multiple soluble factors, like immunomodulation factors [9], angiogenic factor [10], antiapoptotic factors [11], antioxidative factors [12], cell migration factors [13], and so on [14]. Previous studies [15, 16] have shown that vascular endothelial growth factor (VEGF), an important angiogenesis factor, is one of the main soluble factors released by MSC. However, the precise role of VEGF-releasing character of MSC in ARDS remains unknown. Hence, in this study, we aimed to display the role of VEGF-expressing character of MSC in lipopolysaccharide- (LPS-) induced lung injury in rats.

## 2. Materials and Methods

### 2.1. Ethics Statement

Male wild-type SD rats (Laboratory Animal Center, Shanghai, China) were maintained under specific pathogen-free condition (Animal Center, Nanjing, China). All experiments referring to the use of animals were approved by the Committee of Animal Care and Use of Southeast University.

### 2.2. Rat Mesenchymal Stem Cells and Cell Culture

Rat MSC (Cyagen Bioscience, Inc., Guangzhou, China) was derived from normal rat bone marrow. Identification of rat MSC, including the cell surface phenotypes and multipotency, was performed by the supplier. Rat MSC expressed CD90, CD44, and CD29, but not CD34, CD45, and CD11b/c. This characterization was performed by fluorescence-activated cell sorting (FACS) analysis. The abilities of MSC to differentiate into the adipogenic, osteogenic, and chondrogenic lineages were determined by staining with oil red-O, alizarin red, or alcian blue, respectively, after culturing in adipogenic, osteogenic, or chondrogenic differentiation media supplied by Cyagen Bioscience, Inc., for 2-3 weeks.

The culture medium and subculture kits for rat MSC were purchased from Cyagen Bioscience, Inc. Briefly, the MSC culture medium was made of SD rat MSC basal medium supplemented with 10% SD rat MSC-qualified fetal bovine serum, 1% penicillin-streptomycin, and 1% glutamine. MSC used for* in vitro* studies were at the 10th passage after lentivirus transduction. MSC of passage number <9 were used in the rat experiments.

### 2.3. Lentiviral Vectors Mediated VEGF Gene Knockdown in MSC and GFP Reporter Gene Detection

MSC of passage number <6 were used for VEGF gene knockdown experiment. Lentivirus carrying either GFP (LV3-GFP) or both GFP and ShRNA VEGF (LV3-ShRNA VEGF) was offered by GenePharma (Shanghai, China). MSC were cultured in a 6-well plate (Corning, Inc., NY). After 24 hours, we changed the culture medium, added LV3-GFP or LV3-ShRNA VEGF, and maintained the cells in a humidified incubator with 5% CO_2_ at 37°C. After 24 hours, the old medium was aspirated and fresh culture medium was added. Then the cultures were performed by medium change every 2-3 days. The transduction efficiency of the lentiviral vectors in passage 10 transduced MSC was identified using fluorescence microscopy (Olympus Co., Tokyo, Japan).

### 2.4. RNA Isolation and Quantitative Real-Time Polymerase Chain Reaction (qRT-PCR)

MSC treated by LV3-GFP (MSC-GFP) or LV3-ShRNA VEGF (MSC-ShVEGF) were obtained and cultured in cell culture medium, respectively. Total RNA was isolated from MSC, MSC-GFP, or MSC-ShVEGF at the 10th passage, using TRIzol reagent (Takara Bio, Inc., Kyoto, Japan) according to the manufacturer's instruction. RNA was reverse transcribed to single-stranded cDNA using cDNA synthesis reagents (GenePharma, Shanghai, China). Primers used for the RT-PCR were rat VEGF and GAPDH and were supplied by GenePharma (Shanghai, China). The forward and reverse primer sequences for GADPH were 5′-GTGCTGAGTATGTCGTGGAGTCT-3′ and 5′-GGAAGGGGCGGAGATGA-3′. The forward and reverse primer sequences for VEGF were 5′-GAGCAGGAGCCGAAGCC-3′ and 5′-GAGCCCAGAAGTTGGACGA-3′. The RT-PCR assays were performed following the instruction for One-Step RT-PCR described by FUNGLYN BIOTECH INC. (Shanghai, China).

### 2.5. Western Blotting Analysis

MSC, MSC-GFP, or MSC-ShVEGF at the 10th passage were collected after transduction with lentivirus. Total cellular proteins from MSC, MSC-GFP, or MSC-ShVEGF were extracted and separated with the use of SDS-PAGE gels (8%) made by the same way as we previously described [17]. Then, proteins were incubated with primary antibodies to VEGF (1 : 1000 dilution, Abcam Ltd., Cambridge, UK) or GAPDH (1 : 1000 dilution, Cell Signaling Technology, Beverly, MA, USA). The blots were washed three times with tris-1 buffer (Biosharp Biotechnology, Hefei, China) at pH 7.4 containing 0.1% Tween 20 (Sinopharm Chemical Reagent Co., Ltd., Shanghai, China) (TBST) and then incubated with goat anti-rabbit IgG conjugated with horseradish peroxidase (Zhongshan Golden Bridge Biotechnology Co., Ltd., China). Immunoreactive complexes were visualized using chemiluminescence reagents (Thermo Scientific).

### 2.6. Evaluation of VEGF Levels in the Cell Culture Medium by ELISA

MSC, MSC-GFP, and MSC-ShVEGF were seeded in a 12-well plate, respectively, at a density of 1 × 10^5^ cells per well. After 12 hours, the old culture medium was replaced by fresh culture medium. MSC were maintained in an incubator at 37°C, 5% CO_2_ for 24 h. Then the culture media of MSC, MSC-GFP, and MSC-ShVEGF were collected and centrifuged at 3000 rpm for 20 min. The supernatants were collected and stored in −80°C. The levels of VEGF protein in the medium were measured using ELISA kits (ExCellBio, Shanghai, China) according to the manufacturer's instruction.

### 2.7. *In Vitro* Scratch Assay

MSC were seeded in six-well culture plates. When the cells achieved approximately 100% confluence, a scratch was made with a 10 *μ*L sterile pipette tip. The cells were cultured in low-serum (1%) MDEM/12 for an additional 12 h. The images of the wound healing were recorded by a light microscope at 0 h and 12 h after scratching. The horizontal migration abilities of MSC were quantified by measuring the wound widths of five different wound surfaces in each group. The experiment was repeated three times.

### 2.8. Transwell Migration Assay

The vertical migration abilities of MSC were assessed by Transwell migration assay. MSC were seeded on Transwell inserts (6.5 mm diameter and 8 *μ*m pore size; Millipore) at a density of 1 × 10^4^ per insert with 100 *μ*L serum-free culture medium. 600 *μ*L complete cell culture medium was added to the lower chambers. The cells were allowed to migrate at 37°C in a humidified CO_2_ incubator for 10 h. The cells remaining on the upper surface of the insert were removed with cotton swabs. Then, the cells that migrated to the lower surface were fixed by 1% paraformaldehyde for 10 min and then stained with crystal violet (Beyotime Institute of Biotechnology, Haimen, China) for 20 min. The stained cells from five randomly chosen fields were counted under a light microscope.

### 2.9. LPS-Induced ALI in Rats

Six-to-eight-week-old wild-type SD rats (weighing 200 to 250 g) were challenged by LPS (2 mg/kg,* Escherichia coli* 0111:B4, Sigma-Aldrich, St. Louis, MO, USA) dissolved in 100 *μ*L of phosphate buffered saline (PBS, Wisent, Inc., Saint-Bruno, Quebec, Canada) to induce ALI via intratracheal instillation, followed by PBS (100 *μ*L), MSC, MSC-GFP, and MSC-ShVEGF (5 × 10^6^ cells resuspended in 100 *μ*L PBS) infusion via tail vein injection five hours after LPS challenge. Rats (without LPS challenge) that received 100 *μ*L PBS infusion via the tail vein at the same time when other rats received cells treatment were used as a control. Rats were sacrificed at 1 hour, 6 hours, and 24 hours after PBS or cells (MSC, MSC-GFP, and MSC-ShVEGF) infusion. Lung lobes were obtained for further analysis.

### 2.10. Assessment of Lung Edema

As we described previously [18], lung wet weight and body weight were obtained from each group (control, ALI + PBS, ALI + MSC, ALI + MSC-GFP, and ALI + MSC-ShVEGF groups). The lung-wet-weight-to-body-weight ratio (LWW/BW) was measured to determine the lung vascular permeability and lung edema.

### 2.11. Evans Blue Dye Leakage Assay

To evaluate the protein permeability of pulmonary microvascular endothelium, Evans blue dye (Sigma-Aldrich, St. Louis, MO, USA) was used. For the control, ALI, MSC, MSC-GFP, and MSC-ShVEGF groups, Evans blue dye (20 mg/kg) in 1 mL saline was injected into rats via the tail vein at 1 hour, 6 hours, and 24 hours after MSC infusion. 30 minutes later, 100 mL heparinized saline was infused to the right ventricle of the heart to clean up the dye in the lung vascular system and then the whole lung was obtained. 100 mg lung tissue from the right lobe was incubated in formamide (Sigma-Aldrich, St. Louis, MO, USA) for 24 h at 60°C. Then, the concentration of Evans blue dye in lung tissue was measured by a spectrophotometer at 630 nm.

### 2.12. Immunofluorescence

To observe any changes of VE-cadherin expression in the lung, rat lungs were collected gently and then fixed in 4% paraformaldehyde at 4°C. 24 hours later, lung tissues were embedded with optimal cutting temperature medium (OCT, Sakura Finetek USA, Inc., Torrance, CA, USA) and then frozen in −80°C. 24 hours later, lung tissues were cut into 5 *μ*m thick sections. VE-cadherin was stained with anti-VE-cadherin primary antibody (Santa Cruz Biotechnology, Inc., Santa Cruz, CA, USA) and followed by a second antibody Fluorescein- (FITC-) AffiniPure Donkey Anti-Rabbit IgG (H + L) (Jackson ImmunoResearch Inc., PA, USA). The nuclei were stained with 4,6-diamidino-2-phenylindole (DAPI, Sigma-Aldrich). Fluorescence was monitored by an Olympus IX71 microscope (Olympus Co., Tokyo, Japan).

### 2.13. TUNEL Assay

To evaluate the apoptosis of endothelial cells in the lung, the right lung lobes were collected and fixed in 4% paraformaldehyde at 4°C for 24 hours. Then lung tissues were embedded with paraffin and cut into 5 *μ*m thick sections. The sections were then stained by TUNEL Apoptosis Assay Kit (Nanjing Lufei Biotechnology Co., Ltd., Nanjing, China). The numbers of apoptotic lung endothelial cells and total lung endothelial cells were obtained with a microscope. The lung endothelial cells apoptosis index, defined as apoptotic lung endothelial cells number in total lung endothelial cells number, was used to reflect the severity of lung endothelial cells apoptosis. The endothelial cells were quantified by a pathologist based on the images of five randomly selected high-power fields (400×).

### 2.14. Lung Histopathology

To evaluate the severity of lung injury, the right lobe lungs were obtained at 1 hour, 6 hours, and 24 hours after MSC infusion. The lung tissues were then fixed in 4% paraformaldehyde for 24 hours. After fixation, lung tissues were embedded in paraffin and cut into 5 *μ*m sections. Then the sections were stained with a hematoxylin and eosin staining kit purchased from Beyotime Institute of Biotechnology. The severity of lung injury was assessed using lung injury score as we previously described [18].

### 2.15. VEGF Levels in the Lung

The left lung was obtained after rat sacrifice at different time points. The lung tissue was immediately homogenized and centrifuged at 3000 rpm for 20 min. The supernatants were collected and stored at −80°C. The levels of VEGF in the supernatants were measured by ELISA kits (ExCellBio, Shanghai, China) according to the instruction recommended by the manufacturer.

### 2.16. Measurement of Cytokines in the Lung

The left lobe lung was collected and gently homogenized in PBS. Then, the collections were centrifuged at 3000 rpm for 20 min. The supernatants were collected and stored in −80°C. The concentrations of IL-1*β* and IL-10 in the lung were evaluated by ELISA kits (NeoBioscience, Shenzhen, China) according to the manufacturer's instruction.

### 2.17. Statistical Analysis

Data are shown as the means ± standard deviations (SD). Comparisons between two groups were made using unpaired Student's *t*-test. For the comparison between multiple groups, one-way ANOVA with Bonferroni's post hoc test was used. Statistical analyses were performed using SPSS 16.0 software. A value of *p* < 0.05 was considered to be statistically significant.

## 3. Results

### 3.1. Assessment of Lentiviral Vector Mediated VEGF Gene Knockdown

**Figure 1:**
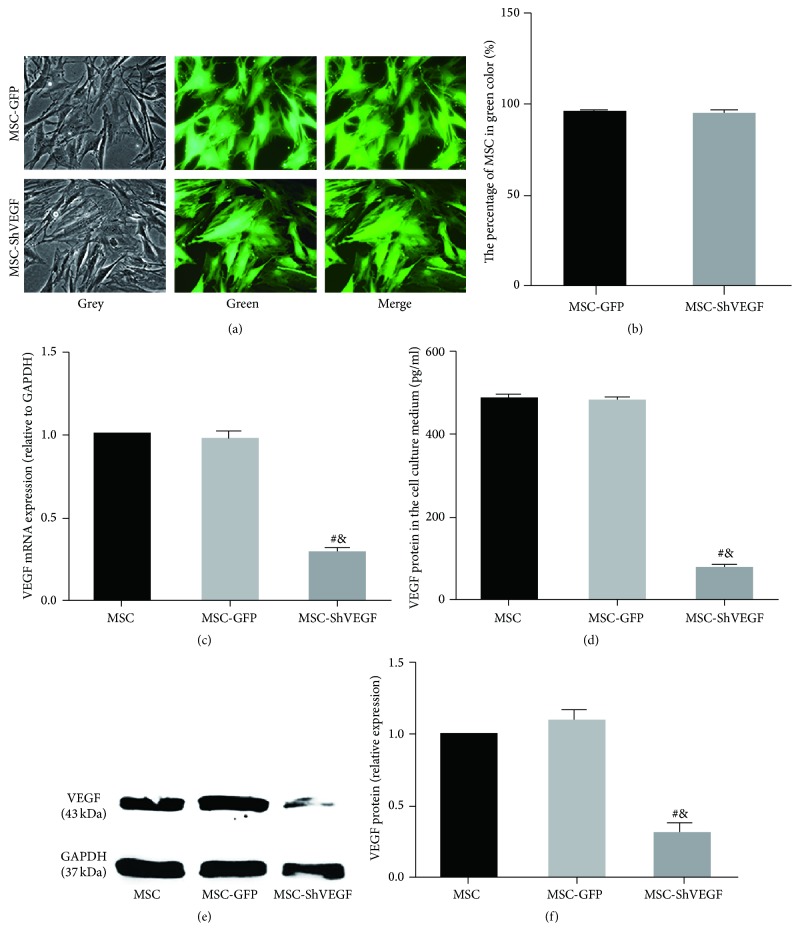
Assessment of lentiviral vectors mediated VEGF gene knockdown. (a) Assessment of the efficiency of lentivirus transduction using fluorescence microscopy, ×400. (b) The quantitative analysis for lentiviral transduction. (c) Evaluation of VEGF mRNA expression in the MSC, MSC-GFP, and MSC-ShVEGF groups. (d) Detection of VEGF protein levels in the culture medium of the MSC, MSC-GFP, and MSC-ShVEGF groups, respectively. (e) Image for the measurement of VEGF protein expression in the MSC, MSC-GFP, and MSC-ShVEGF groups by western blotting analysis. (f) The quantitative result for western blotting analysis (green, GFP; *n* = 3; ^#^ *p* < 0.05 versus MSC group and ^&^ *p* < 0.05 versus MSC-GFP group).

The expression of GFP in the MSC was monitored by fluorescence microscopy, which reflected the transduction efficiency of target gene in the MSC. As Figures 1(a) and 1(b) showed, near 95% MSC were in green color after transduction, indicating a high transduction efficiency of lentiviral vector mediated VEGF gene knockdown. VEGF mRNA expression in the rat MSC, MSC-GFP, and MSC-ShVEGF at the 10th passage after lentiviral transduction was determined by quantitative real-time PCR. According to the result, VEGF mRNA levels were significantly lower in the MSC-ShVEGF group than that in the MSC (^#^ *p* < 0.05) or MSC-GFP (^&^ *p* < 0.05) group. There was no statistical difference in the VEGF mRNA levels between the MSC and MSC-GFP groups (*p* > 0.05) (Figure 1(c)).

The data from western blotting analysis showed that the VEGF protein expression in the cytoplasm was also decreased dramatically in the MSC-ShVEGF group (Figures 1(e) and 1(f)), compared with the MSC group (^#^ *p* < 0.05) or MSC-GFP group (^&^ *p* < 0.05). In addition, the result from ELISA showed that VEGF protein levels in the culture medium of MSC-ShVEGF were statistically lower than that in the MSC (^#^ *p* < 0.05) or MSC-GFP (^&^ *p* < 0.05) (Figure 1(d)) culture medium. However, no significant difference was found between the MSC and MSC-GFP groups in the VEGF protein levels in both the cytoplasm and cell culture medium. These results suggested that VEGF gene knockdown mediated by lentiviral vectors is efficient and relatively stable.

### 3.2. The Influence of VEGF Gene Knockdown on the Migration Property of MSC

**Figure 2:**
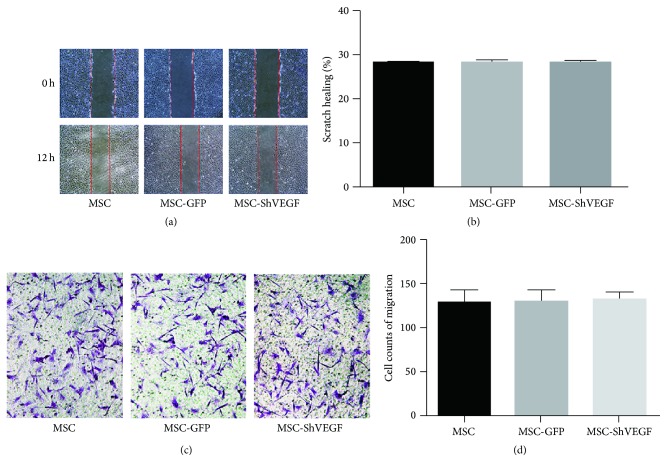
The effect of VEGF gene knockdown on the migration property of MSC. (a) The horizontal migration ability of the MSC with VEGF gene knockdown was evaluated by the scratch assay. The wound sites (area without cells) were observed and photographed at 0 and 12 hours (×40). (b) The bar graph showed the quantitative results of scratch healing. (c) The vertical migration ability of the MSC with VEGF gene knockdown was evaluated by Transwell migration assay. The migrating cells on the lower surface of the Transwell inserts were fixed by 4% paraformaldehyde and stained with crystal violet and observed under the microscope, ×200. (d) The bar graph showed the quantitative results of cell migration (*n* = 3).

To evaluate the influence of VEGF gene knockdown on the migration property of MSC, the scratch assay and Transwell migration assay were performed. As shown in Figures 2(a) and 2(b), no significant difference was found in the wound width which reflected the horizontal migration ability of MSC between the MSC group and MSC-GFP or MSC-ShVEGF group. The vertical migration ability of MSC was assessed by Transwell migration assay. No statistical difference was shown in the vertical migration ability between the MSC group and MSC-GFP or MSC-ShVEGF group (Figures 2(c) and 2(d)). Taken together, both the horizontal and vertical migration properties of MSC were not changed after the VEGF gene knockdown, suggesting that the MSC-ShVEGF would have the same chance to migrate to injured sites as wild MSC.

### 3.3. The Effect of MSC with or without VEGF-Expressing Action on Pulmonary Vascular Permeability in Rats with LPS-Induced ALI

**Figure 3:**
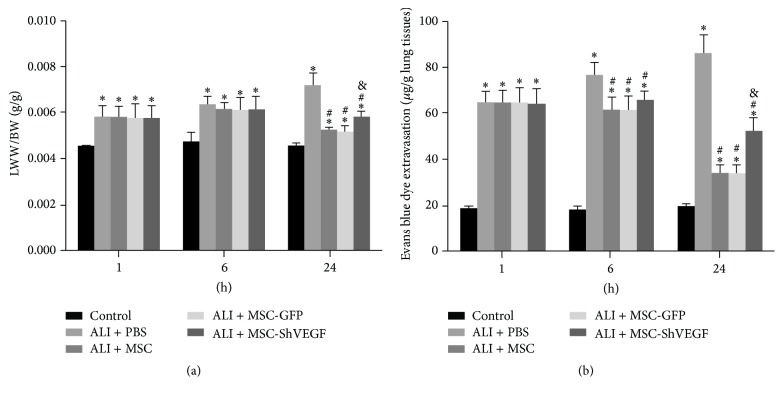
The effects of MSC, MSC-GFP, or MSC-ShVEGF on the pulmonary vascular permeability in rats with LPS-induced ALI. (a) A comparison of the lung-wet-weight-to-body-weight ratios (LWW/BW) in different groups at 1, 6, and 24 hours after cells injection. (b) The quantitative analysis of Evans blue dye leakage at 1, 6, and 24 hours after cells injection (*n* = 6; ^*∗*^ *p* < 0.05 versus control group, ^#^ *p* < 0.05 versus ALI + PBS group, and ^&^ *p* < 0.05 versus ALI + MSC group).

To examine the effect of MSC with or without VEGF-expressing action on the pulmonary vascular permeability, LWW and BW of the rats were obtained. Besides, Evans blue dye extravasation was carried out to assess the large molecular permeability of lung vascular system. As Figure 3 showed, LWW/BW in the ALI + PBS group was increased dramatically at 1 hour, 6 hours, and 24 hours (^*∗*^ *p* < 0.05) after PBS treatment. After treatment with the MSC, MSC-GFP, or MSC-ShVEGF, LWW/BW was decreased significantly at 24 hours (^#^ *p* < 0.05), indicating that MSC had a beneficial effect on pulmonary vascular permeability in LPS-induced lung injury in rats. However, LWW/BW levels in rats treated by MSC with VEGF gene knockdown were significantly higher than that in rats treated by the MSC or MSC-GFP (^&^ *p* < 0.05), demonstrating that VEGF-expressing character of MSC played an important role in protecting pulmonary vascular permeability.

Similarly, LPS stimulation increased Evans blue dye extravasation from lung vascular to lung interstitial and alveolar space at 1 hour, 6 hours, and 24 hours (^*∗*^ *p* < 0.05) after PBS injection. After treatment with the MSC, MSC-GFP, or MSC-ShVEGF, Evans blue dye extravasation was decreased statistically at 6 hours and 24 hours (^#^ *p* < 0.05). This result was different from that of LWW/BW. Evans blue dye extravasation was decreased much earlier (at 6 hours) than LWW/BW after MSC treatment, disclosing that MSC may have a much stronger beneficial effect on protein permeability. Besides, Evans blue dye extravasation in rats treated by the MSC-ShVEGF was significantly higher than that in rats treated by the MSC or MSC-GFP (^&^ *p* < 0.05) at 24 hours, suggesting that MSC can improve lung vascular permeability impaired by LPS and this beneficial effect of MSC on lung vascular permeability was associated with the ability of MSC to release VEGF.

### 3.4. Detection of VE-Cadherin Expression in the LPS Injured Lung after MSC or MSC-ShVEGF Treatment Using Immunofluorescence

**Figure 4:**
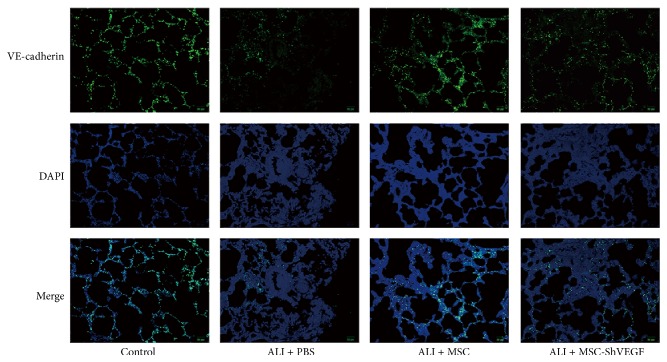
Detection of the changes of VE-cadherin in the LPS injured lung after the MSC or MSC-ShVEGF injection using immunofluorescence. The intercellular junction in the LPS injured lung was evaluated by detection of the expression of adherens junction protein VE-cadherin using immunofluorescence 24 hours after the MSC or MSC-ShVEGF treatment (×200; blue, DAPI; green, VE-cadherin).

VE-cadherin is one of adherens junction proteins, which plays a critical role in the paracellular permeability of pulmonary vascular endothelium. To investigate the effect of MSC with VEGF gene knockdown on intercellular junctions in rat lung stimulated with LPS, the expression of VE-cadherin was observed using immunofluorescence at 24 hours after cells treatment. As Figure 4 showed, the VE-cadherin expression in the ALI + PBS group was decreased markedly after PBS infusion at 24 hours. After treatment with the MSC or MSC-ShVEGF, the VE-cadherin expression was increased dramatically comparing with the ALI + PBS group. However, the increase in the VE-cadherin expression in the ALI + MSC-ShVEGF group was significantly lower than that in the ALI + MSC group. These results suggested that MSC protected the cell adherens junction in the injured lung and VEGF-expressing character was one explanation for this protective effect of MSC.

### 3.5. Assessment of Endothelial Cells Apoptosis in the LPS Injured Lung after the MSC or MSC-ShVEGF Treatment Using TUNEL Assay

**Figure 5:**
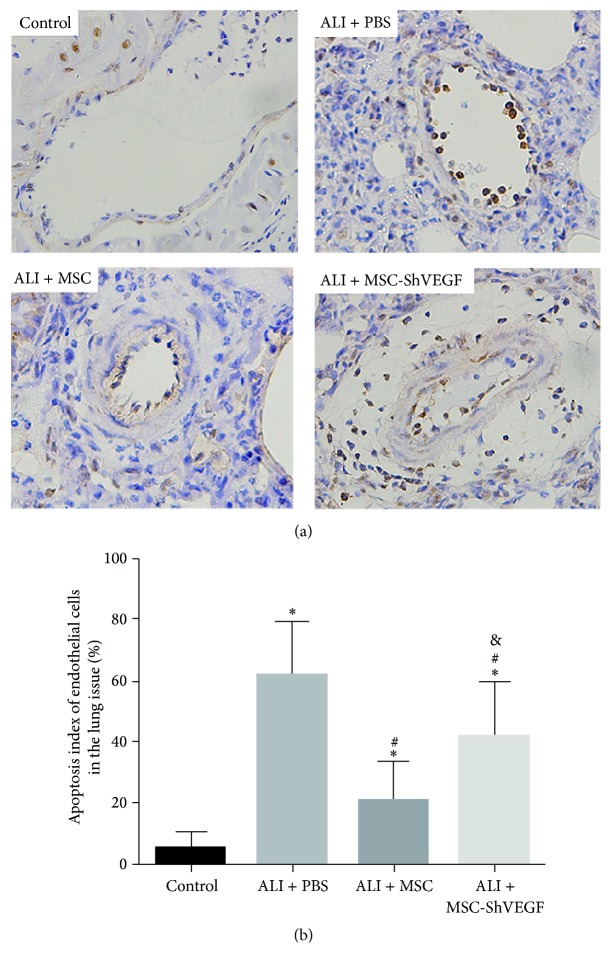
Evaluation of the apoptosis of pulmonary vascular endothelial cell in the LPS injured lung after the MSC or MSC-ShVEGF infusion by TUNEL assay. (a) Representative images of the apoptosis of lung endothelial cells in the LPS injured lung in different groups at 24 hours after the MSC treatment (×400). (b) The quantitative result of the apoptosis index of lung endothelial cell in the LPS injured lung in different groups at 24 hours after MSC injection (*n* = 3; ^*∗*^ *p* < 0.05 versus control group, ^#^ *p* < 0.05 versus ALI + PBS group, and ^&^ *p* < 0.05 versus ALI + MSC group).

Endothelial cell apoptosis will compromise endothelial layer integrity and lead to the increase in pulmonary vascular permeability. In the current study, the effect of the MSC or MSC-ShVEGF on the endothelium apoptosis was evaluated using a TUNEL assay at 24 hours after cells infusion. As Figure 5 showed, the apoptosis index of the lung endothelium increased after LPS challenge. With MSC treatment, it decreased to a large degree. However, in the ALI + MSC-ShVEGF group, the apoptosis index of the lung endothelium remained significantly high. This result indicated that MSC protected lung endothelial cells from apoptosis in ALI and this protective effect was related to the ability of MSC to secrete VEGF.

### 3.6. Evaluation of the Effect of MSC with or without VEGF-Expressing Action on ALI by Histopathology

**Figure 6:**
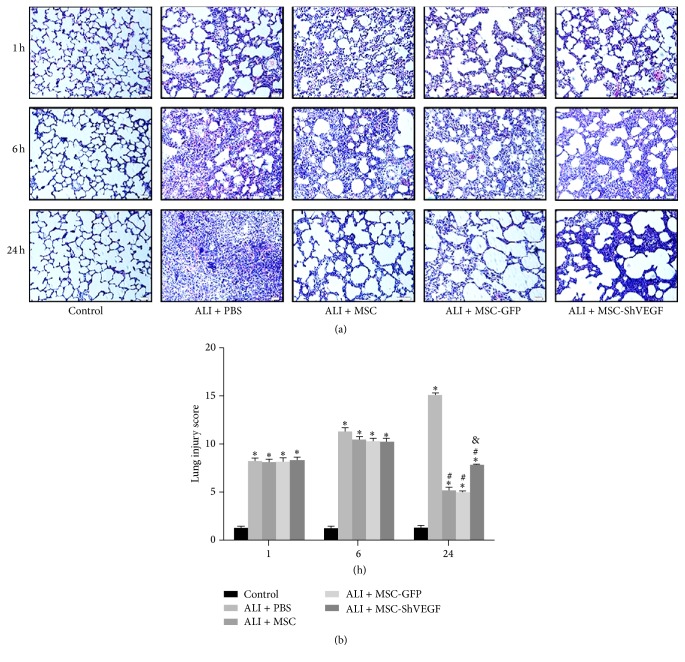
Evaluation of the histopathology of the LPS injured lung after the MSC, MSC-GFP, or MSC-ShVEGF treatment. (a) Representative H&E staining images (×100) of lung sections in each group at 1, 6, and 24 hours. (b) The quantitative analysis of the lung injury scores in each group at different time points (*n* = 6; ^*∗*^ *p* < 0.05 versus control group, ^#^ *p* < 0.05 versus ALI + PBS group, and ^&^ *p* < 0.05 versus ALI + MSC group).

The image of lung histopathology (Figure 6) revealed extensive leukocytes infiltrates in the lung, diffuse interstitial and alveolar edema, remarkable interalveolar septal thickening, lung hemorrhage, hyaline membrane formation in alveolar space, and alveolar collapse after LPS stimulation. The lung injury score was around 8.23 at 1 hour but increased to 11.27 at 6 hours and 15.15 at 24 hours after PBS injection in the ALI + PBS group. The administration of the MSC, MSC-GFP, or MSC-ShVEGF attenuated lung injury as shown by histopathology as well as the lung injury score at 24 hours (^#^ *p* < 0.05). However, in the ALI + MSC-ShVEGF group, the lung injury score was higher than that in the ALI + MSC or ALI + MSC-GFP group (^&^ *p* < 0.05). There was no significant difference in lung injury score between the ALI + MSC and ALI + MSC-GFP groups. These results disclosed that VEGF gene knockdown in MSC significantly diminished the therapeutic effect of MSC on LPS-induced ALI.

### 3.7. The Role of VEGF-Expressing Character of MSC in Cytokine Levels in the LPS Injured Lung

**Figure 7:**
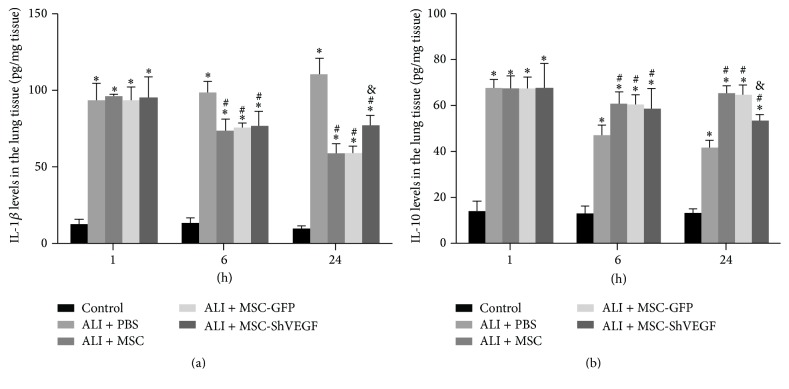
The cytokine levels in the LPS injured lung after the MSC, MSC-GFP, or MSC-ShVEGF treatment by ELISA. (a) IL-1*β* levels in the LPS injured lung in different groups at 1, 6, and 24 hours after cells treatment measured by ELISA. (b) IL-10 levels in the LPS injured lung in different groups at 1, 6, and 24 hours after cells treatment measured by ELISA (*n* = 6; ^*∗*^ *p* < 0.05 versus control group, ^#^ *p* < 0.05 versus ALI + PBS group, and ^&^ *p* < 0.05 versus ALI + MSC group).

We also investigated the effect of the MSC-GFP and MSC with VEGF gene knockdown on the cytokine IL-10 and IL-1*β* levels in the lung. As Figure 7(a) showed, the lung IL-1*β* levels were increased markedly at 1 hour, 6 hours, and 24 hours (^*∗*^ *p* < 0.05) in the ALI + PBS group. After treatment with the MSC, MSC-GFP, and MSC-ShVEGF, IL-1*β* levels were decreased significantly at 6 hours and 24 hours (^#^ *p* < 0.05). No significant difference was observed between the ALI + MSC-ShVEGF group and ALI + MSC or ALI + MSC-GFP group at 6 hours in lung IL-1*β* levels. However, IL-1*β* levels in the ALI + MSC-ShVEGF group were higher than that in the ALI + MSC or ALI + MSC-GFP group at 24 hours (^&^ *p* < 0.05). For IL-10 expression in the lung tissue, data showed that it was decreased significantly after PBS infusion in the ALI + PBS group at 1 hour, 6 hours, and 24 hours (^*∗*^ *p* < 0.05). However, it was increased markedly after the MSC, MSC-GFP, or MSC-ShVEGF treatment at 6 hours and 24 hours. In addition, compared with the ALI + MSC or ALI + MSC-GFP group, IL-10 levels were significantly lower in the ALI + MSC-ShVEGF group. These results indicated that MSC can control the inflammation in the lung induced by LPS and this effect of MSC was related to its property to release VEGF.

### 3.8. The Effect of VEGF-Expressing Character of MSC on VEGF Levels in the LPS Injured Lung

**Figure 8:**
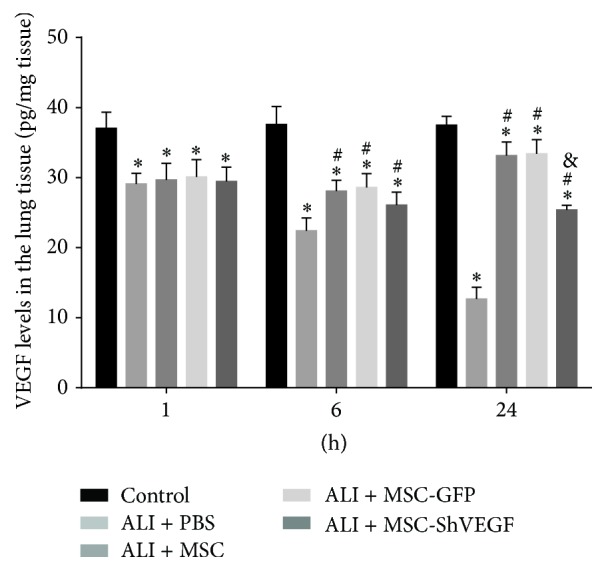
VEGF expression in the LPS injured lung after the MSC, MSC-GFP, or MSC-ShVEGF treatment. VEGF protein levels in the LPS injured lung from different groups at 1, 6, and 24 hours after cells delivery measured by ELISA (*n* = 6; ^*∗*^ *p* < 0.05 versus control group, ^#^ *p* < 0.05 versus ALI + PBS group, and ^&^ *p* < 0.05 versus ALI + MSC group).

To learn the effect of MSC infusion on the VEGF levels in ALI rats, we measured the VEGF expression in the lung. As Figure 8 showed, VEGF protein levels in the lung decreased gradually after LPS challenge. MSC treatment raised the decreased VEGF levels induced by LPS at 6 hours and 24 hours. Interestingly, VEGF levels in the ALI + MSC-ShVEGF group were significantly lower compared with the ALI + MSC group or ALI + MSC-GFP group at 24 hours. The results indicated that MSC maintained the VEGF level in the injured lung and VEGF-secreting action in MSC played a critical role in the maintenance of the VEGF expression in the injured lung.

## 4. Discussions

Recently, more and more studies have shown that MSC has a strong paracrine capacity and proposed it as the principal mechanism that contributes to repair the injured organs [8, 19]. In the growth medium of murine marrow derived MSC, a great amount of VEGF has been found by Kinnaird et al. [16], indicating a VEGF-expressing character of MSC. Moreover, the medium has enhanced proliferation of endothelial cells in a dose-dependent manner and anti-VEGF antibodies partly attenuated this effect, suggesting that the VEGF released by MSC is biologically active. VEGF is also identified in the culture medium of human MSC by Crisostomo et al. [15]. Besides, they have also found that LPS and hypoxia increased the VEGF releasing of MSC.

It is widely known that VEGF plays a vital role in vascular network system remodeling. VEGF is an important mitogen that regulates endothelial cell survival, differentiation, angiogenesis, the recruitment of endothelial progenitor cells, and so on [20]. It has been shown to be critical for the structure maintenance and functional homeostasis of the normal lung [20]. Furthermore, Song et al. [21] have suggested that VEGF may contribute to vascular endothelial repair and function as a protective factor against ALI. However, it has also been reported that increasing VEGF level could lead to an increase in capillary leakage and therefore may lead to a much worse outcome [22]. Madonna et al. [23] have reported that adipose tissue derived MSC conjugated with VEGF-releasing microcarriers promotes repair in murine myocardial infarction. However, little is known about the role of the VEGF-expressing character of MSC in the therapeutic effect of MSC on ALI/ARDS.

In this study, we firstly constructed the VEGF gene deficient MSC using lentiviral vectors transduction and then detected the VEGF mRNA expression as well as the protein expression in the cytoplasm and cell culture medium. The results showed that both the VEGF mRNA and protein expression of MSC-ShVEGF at the 10th passage after transduction were dramatically reduced, suggesting that lentivirus mediated VEGF gene knockdown in the MSC was efficient and stable. Moreover, we also assessed the migration ability of the MSC-ShVEGF. The results from the Transwell migration assay and scratch assay showed that both the vertical and horizontal migration abilities were not changed significantly compared with the normal MSC, which promised the MSC-ShVEGF the same ability to home to the injured tissue as the normal MSC. Therefore, we successfully obtained the VEGF deficient MSC through lentiviral transduction. It facilitated our further* in vivo* study.

Furthermore, our* in vivo* study showed that MSC itself had a protective effect on lung permeability and this was associated with the VEGF-expressing action of MSC. MSC infusion reduced the increase in the LWW/BW and Evans blue dye extravasation induced by LPS, reflecting an improvement in lung permeability. Moreover, the loss of intercellular junction protein VE-cadherin in the lung tissue and apoptosis index of lung endothelial cell were also decreased after the MSC treatment, suggesting that MSC played a positive role in maintaining the integrity of lung endothelium. However, these protective effects of MSC were diminished partly but significantly after the VEGF gene was knocked down in the MSC. Besides, MSC treatment significantly reduced the proinflammatory cytokine IL-1*β* levels and elevated the anti-inflammatory cytokine IL-10 levels in the lung tissue as shown in this study, suggesting that MSC corrected the imbalance of lung inflammation induced by LPS. MSC was widely reported to have an anti-inflammatory action in numerous studies [24–26]. Here our results were consistent with those previous studies. However, as shown in Figure 7, these beneficial effects of MSC on inflammation were reduced in the MSC-ShVEGF treated rats. Our result from histopathology also indicated a protective effect of the MSC with VEGF-expressing action on ALI/ARDS. In conclusion, VEGF-expressing character is required for MSC to exert a therapeutic effect on ALI/ARDS.

The underlying mechanism for the requirement of VEGF-expressing character of MSC in the treatment of ALI/ARDS may be related to the VEGF expression in the lung. Therefore, we detected the VEGF levels in the lung tissue as shown in Figure 8. VEGF expression in the lung was decreased after LPS challenge. MSC treatment increased VEGF to the level of a normal lung. However, VEGF gene knockdown diminished this beneficial effect of MSC on VEGF expression in the lung. VEGF has been proved to have angiogenic and antiapoptosis properties [27]. It is a critical survival factor for both the endothelial and epithelial cells in the lung. Studies have shown that VEGF accelerated restoration of endothelial damage [20, 28]. Except for the protective effects of VEGF on the endothelium, VEGF has also been reported to stimulate growth of epithelial cells [29] and surfactant production [30]. At high concentrations VEGF may inhibit alveolar epithelial type 2 cell (AE2) apoptosis [29] and accelerate the restoration of epithelial damage [31]. Although there are some studies reporting that a high VEGF level may be harmful and sometimes indicate a lower chance of survival since it leads to pulmonary edema and increased lung vascular permeability [32], insufficient VEGF level is also shown to be disadvantageous for the development and restoration of the lung [33, 34].

There are studies showing that bioactive VEGF is reduced in the epithelial lining fluid (ELF) of patients with pulmonary diseases including ALI/ARDS [35]. Moreover, the early restoration of ELF VEGF levels is associated with recovery from lung injury in ARDS. It has been suggested that VEGF within the lung may therefore play a critical role in contributing to the regulation of alveolar-capillary permeability and promoting lung repair [36]. Our results are consistent with these previous ideas that VEGF at a “sufficient” level is required for promoting lung restoration. VEGF-expressing character of MSC plays an important role in maintaining a “sufficient” VEGF level in the lung tissue in the MSC treatment for ALI/ARDS.

There are several limitations in this study. Firstly, we only detected the adherens junction protein VE-cadherin. No tight junctions or cytoskeletons protein which also plays a very important part in maintaining the integrity of the lung endothelium was observed. Secondly, we have assessed the VEGF levels in the lung after MSC infusion and found that it increased significantly at 24 h compared with the ALI group. However, numerous cell types around the airspace [37] can release VEGF. Here, a further experiment is needed to confirm that the increased VEGF was released by MSC or by other cell types in the lung tissue. This may help us to have a much better understanding of the mechanism for the beneficial effect of MSC on ARDS.

## 5. Conclusions

MSC restores lung permeability which may be associated with the decrease in the loss of adherens junction protein VE-cadherin and lung vascular endothelial cell apoptosis, controls the inflammation, and attenuates lung injury in LPS-induced ALI rats in part by maintaining a “sufficient” VEGF level in the injured lung. Moreover, the VEGF-expressing character of MSC plays a positive role in these beneficial effects of MSC. These results indicate a new mechanism for the therapeutic effect of MSC on ALI/ARDS.

## References

[1] (2000). Ventilation with lower tidal volumes as compared with traditional tidal volumes for acute lung injury and the acute respiratory distress syndrome. *The New England Journal of Medicine*.

[2] Wiedemann H. P., Wheeler A. P., Bernard G. R. (2006). Comparison of two fluid-management strategies in acute lung injury. *The New England Journal of Medicine*.

[3] Guérin C., Reignier J., Richard J.-C. (2013). Prone positioning in severe acute respiratory distress syndrome. *The New England Journal of Medicine*.

[4] Hu S. L., He H. L., Pan C. (2014). The effect of prone positioning on mortality in patients with acute respiratory distress syndrome: a meta-analysis of randomized controlled trials. *Critical Care*.

[5] Papazian L., Forel J.-M., Gacouin A. (2010). Neuromuscular blockers in early acute respiratory distress syndrome. *The New England Journal of Medicine*.

[6] Buregeya E., Fowler R. A., Talmor D. S., Twagirumugabe T., Kiviri W., Riviello E. D. (2014). Acute respiratory distress syndrome in the global context. *Global Heart*.

[7] Ware L. B., Matthay M. A. (2000). The acute respiratory distress syndrome. *The New England Journal of Medicine*.

[8] Liang X., Ding Y., Zhang Y., Tse H.-F., Lian Q. (2014). Paracrine mechanisms of mesenchymal stem cell-based therapy: current status and perspectives. *Cell Transplantation*.

[9] Ho M. S. H., Mei S. H. J., Stewart D. J. (2015). The immunomodulatory and therapeutic effects of mesenchymal stromal cells for acute lung injury and sepsis. *Journal of Cellular Physiology*.

[10] Liu C., Fan Y., Zhou L. (2015). Pretreatment of mesenchymal stem cells with angiotensin II enhances paracrine effects, angiogenesis, gap junction formation and therapeutic efficacy for myocardial infarction. *International Journal of Cardiology*.

[11] Wang S.-P., Wang Z.-H., Peng D.-Y., Li S.-M., Wang H., Wang X.-H. (2012). Therapeutic effect of mesenchymal stem cells in rats with intracerebral hemorrhage: reduced apoptosis and enhanced neuroprotection. *Molecular Medicine Reports*.

[12] Okazaki T., Magaki T., Takeda M. (2008). Intravenous administration of bone marrow stromal cells increases survivin and Bcl-2 protein expression and improves sensorimotor function following ischemia in rats. *Neuroscience Letters*.

[13] Tang J.-M., Wang J.-N., Zhang L. (2011). VEGF/SDF-1 promotes cardiac stem cell mobilization and myocardial repair in the infarcted heart. *Cardiovascular Research*.

[14] Lee J. W., Fang X., Krasnodembskaya A., Howard J. P., Matthay M. A. (2011). Concise review: mesenchymal stem cells for acute lung injury: role of paracrine soluble factors. *STEM CELLS*.

[15] Crisostomo P. R., Wang Y., Markel T. A., Wang M., Lahm T., Meldrum D. R. (2008). Human mesenchymal stem cells stimulated by TNF-*α*, LPS, or hypoxia produce growth factors by an NF*κ*B- but not JNK-dependent mechanism. *American Journal of Physiology—Cell Physiology*.

[16] Kinnaird T., Stabile E., Burnett M. S. (2004). Local delivery of marrow-derived stromal cells augments collateral perfusion through paracrine mechanisms. *Circulation*.

[17] Liu A.-R., Liu L., Chen S. (2013). Activation of canonical wnt pathway promotes differentiation of mouse bone marrow-derived MSCs into type II alveolar epithelial cells, confers resistance to oxidative stress, and promotes their migration to injured lung tissue in vitro. *Journal of Cellular Physiology*.

[18] He H., Liu L., Chen Q. (2015). Mesenchymal stem cells overexpressing angiotensin-converting enzyme 2 rescue lipopolysaccharide-induced lung injury. *Cell Transplantation*.

[19] Matthay M. A. (2015). Therapeutic potential of mesenchymal stromal cells for acute respiratory distress syndrome. *Annals of the American Thoracic Society*.

[20] Barratt S., Medford A. R., Millar A. B. (2014). Vascular endothelial growth factor in acute lung injury and acute respiratory distress syndrome. *Respiration*.

[21] Song J., Lu H., Zheng X., Huang X. (2015). Effects of vascular endothelial growth factor in recovery phase of acute lung injury in mice. *Lung*.

[22] Zhang L., Liu H., Peng Y.-M., Dai Y.-Y., Liu F.-Y. (2015). Vascular endothelial growth factor increases GEnC permeability by affecting the distributions of occludin, ZO-1 and tight juction assembly. *European Review for Medical and Pharmacological Sciences*.

[23] Madonna R., Petrov L., Teberino M. A. (2015). Transplantation of adipose tissue mesenchymal cells conjugated with VEGF-releasing microcarriers promotes repair in murine myocardial infarction. *Cardiovascular Research*.

[24] Kim Y., Jo S. H., Kim W. H., Kweon O. (2015). Antioxidant and anti-inflammatory effects of intravenously injected adipose derived mesenchymal stem cells in dogs with acute spinal cord injury. *Stem Cell Research & Therapy*.

[25] Pers Y.-M., Ruiz M., Noël D., Jorgensen C. (2015). Mesenchymal stem cells for the management of inflammation in osteoarthritis: state of the art and perspectives. *Osteoarthritis and Cartilage*.

[26] Cruz F. F., Borg Z. D., Goodwin M. (2015). Systemic administration of human bone marrow-derived mesenchymal stromal cell extracellular vesicles ameliorates *Aspergillus* hyphal extract-induced allergic airway inflammation in immunocompetent mice. *Stem Cells Translational Medicine*.

[27] Karihaloo A., Karumanchi S. A., Cantley W. L., Venkatesha S., Cantley L. G., Kale S. (2005). Vascular endothelial growth factor induces branching morphogenesis/ tubulogenesis in renal epithelial cells in a neuropilin-dependent fashion. *Molecular and Cellular Biology*.

[28] Kim Y.-G., Suga S.-I., Kang D.-H. (2000). Vascular endothelial growth factor accelerates renal recovery in experimental thrombotic microangiopathy. *Kidney International*.

[29] Brown K. R. S., England K. M., Goss K. L., Snyder J. M., Acarregui M. J. (2001). VEGF induces airway epithelial cell proliferation in human fetal lung in vitro. *American Journal of Physiology—Lung Cellular and Molecular Physiology*.

[30] Compernolle V., Brusselmans K., Acker T. (2002). Loss of HIF-2*α* and inhibition of VEGF impair fetal lung maturation, whereas treatment with VEGF prevents fatal respiratory distress in premature mice. *Nature Medicine*.

[31] Yancopoulos G. D., Davis S., Gale N. W., Rudge J. S., Wiegand S. J., Holash J. (2000). Vascular-specific growth factors and blood vessel formation. *Nature*.

[32] Kaner R. J., Ladetto J. V., Singh R., Fukuda N., Matthay M. A., Crystal R. G. (2000). Lung overexpression of the vascular endothelial growth factor gene induces pulmonary edema. *American Journal of Respiratory Cell and Molecular Biology*.

[33] Tang K., Rossiter H. B., Wagner P. D., Breen E. C. (2004). Lung-targeted VEGF inactivation leads to an emphysema phenotype in mice. *Journal of Applied Physiology*.

[34] Perkins G. D., Roberts J., McAuley D. F. (2005). Regulation of vascular endothelial growth factor bioactivity in patients with acute lung injury. *Thorax*.

[35] Thickett D. R., Armstrong L., Millar A. B. (2002). A role for vascular endothelial growth factor in acute and resolving lung injury. *American Journal of Respiratory and Critical Care Medicine*.

[36] Mura M., Dos Santos C. C., Stewart D., Liu M. (2004). Vascular endothelial growth factor and related molecules in acute lung injury. *Journal of Applied Physiology*.

[37] Maitre B., Boussat S., Jean D. (2001). Vascular endothelial growth factor synthesis in the acute phase of experimental and clinical lung injury. *European Respiratory Journal*.

